# Application of the Dehydration Homogeneous Liquid–Liquid Extraction (DHLLE) Sample Preparation Method for Fingerprinting of Honey Volatiles

**DOI:** 10.3390/molecules26082277

**Published:** 2021-04-14

**Authors:** Piotr M. Kuś, Igor Jerković

**Affiliations:** 1 Department of Pharmacognosy, Wrocław Medical University, ul. Borowska 211a, 50-556 Wrocław, Poland; 2 Department of Organic Chemistry, Faculty of Chemistry and Technology, University of Split, Ruđera Boškovića 35, 21000 Split, Croatia; igor@ktf-split.hr

**Keywords:** honey chemical markers, volatile profiling, green extraction

## Abstract

Recently, we proposed a new sample preparation method involving reduced solvent and sample usage, based on dehydration homogeneous liquid–liquid extraction (DHLLE) for the screening of volatiles and semi-volatiles from honey. In the present research, the method was applied to a wide range of honeys (21 different representative unifloral samples) to determine its suitability for detecting characteristic honey compounds from different chemical classes. GC-FID/MS disclosed 130 compounds from different structural and chemical groups. The DHLLE method allowed the extraction and identification of a wide range of previously reported specific and nonspecific marker compounds belonging to different chemical groups (including monoterpenes, norisoprenoids, benzene derivatives, or nitrogen compounds). For example, DHLLE allowed the detection of cornflower honey chemical markers: 3-oxo-*retro*-α-ionols, 3,4-dihydro-3-oxoedulan, phenyllactic acid; coffee honey markers: theobromine and caffeine; linden honey markers: 4-isopropenylcyclohexa-1,3-diene-1-carboxylic acid and 4-(2-hydroxy-2-propanyl)cyclohexa-1,3-diene-1-carboxylic acid, as well as furan derivatives from buckwheat honey. The obtained results were comparable with the previously reported data on markers of various honey varieties. Considering the application of much lower volumes of very common reagents, DHLLE may provide economical and ecological advantages as an alternative sample preparation method for routine purposes.

## 1. Introduction

Honey volatiles fingerprinting is one of the most promising methods for honey quality control and classification according to the botanical origin. It is very sensitive and connected to sensory qualities that are important for consumers and their preferences [1,2]. Semi-volatile compounds are also of particular interest, as they may be less vulnerable to differences caused by the technological processing or storage of honey in comparison to highly volatile compounds. Different authors have developed and reviewed a range of methodologies for honey volatiles extraction such as solid-phase extraction (SPE), headspace solid-phase microextraction (HS-SPME), as well as ultrasound solvent extraction (USE) [3,4]. However, different approaches developed for the sample preparation and screening of honey volatiles have various drawbacks when applied for routine analyses. They include the high cost of consumables (SPE, HS-SPME, and SPME), the use of high volumes of solvents such as pentane, diethyl ether, or dichloromethane (USE and SPE), as well as the high selectivity (HS-SPME). Recently, we proposed a new method based on dehydration homogeneous liquid–liquid extraction (DHLLE) for screening volatiles and semi-volatiles from honey that involves reduced solvent and sample usage [5]. The methodology consists of a few steps: the dissolving of honey in water and isopropanol, the isolation of isopropanol extract by dehydration with anhydrous magnesium sulfate, followed by the changing of polarity of the extract by the addition of dichloromethane and the subsequent purification by washing with water, concentrating, and drying the sample prior to GC-MS analysis. Satisfactory recoveries (up to 93.5%) and repeatabilities (RSD up to 8.9%) were obtained for various volatiles/semi-volatiles of different structures and polarities, including monoterpenes, benzene derivatives, or methylxanthines that are common in honeys [5]. The results obtained with DHLLE for apple honey were comparable or better than those obtained with other methods based on ultrasonic extraction with dichloromethane [5]. The amounts of sample and solvents used in DHLLE were much lower than in other available methods, but the repeatabilities and recoveries were maintained at reasonable levels; therefore, it was found to be potentially useful for the routine screening, fingerprinting, and detection of chemical markers in the honey phytochemical profiles. The method allows for the significant reduction of the consumption of reagents in comparison with other methods such as USE or SPE, which makes it more cost-efficient and environmentally friendly [5]. Nevertheless, considering the promising characteristics of the methodology, there is a need to evaluate it on a larger range of honey varieties to prove its suitability for practical applications. Therefore, the scope of the study was to (i) apply the newly developed method (DHLLE) on a wide range of selected unifloral honey samples for the first time, to check its suitability for the detection of characteristic compounds (including specific and nonspecific chemical markers of botanical origin) from different chemical classes (including aliphatic compounds, monoterpenes, norisoprenoids, nitrogen containing compounds, and others); (ii) determine the nontargeted volatile organic compounds (VOCs) chemical profiles of different honey samples with the DHLLE method for the first time and to evaluate the obtained results with the available literature data. In addition, the present research provides a comparison of the VOCs chemical profiles of the 21 different selected unifloral honeys, which were obtained under the same preparative conditions; this is very rare in the literature and makes it a useful resource in the area of authenticity and traceability of varietal honeys.

## 2. Results and Discussion

### 2.1. GC-MS Profiles of the Obtained Honey Extracts

A total of 21 different unifloral honey samples were analyzed, disclosing 130 compounds that may be divided into different structural and chemical groups.

The most numerous group (37 compounds) was benzene derivatives, including simple derivatives, as well as phenylpropanoids. The latter were synthetized from aromatic amino acids, tyrosine and phenylalanine. The most abundant compound was methyl syringate, which was present in most of the samples, ranging from 0.7% to 85.8%, and is the main component of asphodel and savory honey extracts (85.8% and 72.6%, respectively). Other abundant compounds were phenylalanine catabolism products: phenyllactic acid present in cornflower, heather, and purple milk thistle honeys (9.4, 25.6, and 26.5%, respectively); phenylacetic acid present in most of the samples (0.2%–15.6%) and most abundant in fir and dandelion honeys (10.3% and 15.6%, respectively); phenylacetaldehyde present in most of the samples (0.1%–21.0%) and being the most abundant in sage and purple milk thistle honeys (14.3% and 21.0%, respectively).

Another relevant group of compounds present in the investigated samples was the isoprenoid group consisting of 18 monoterpenes and 18 norisoprenoids. Terpenes are produced from 2-isopentenyl pyrophosphate (2-IPP) and its isomer, 3-isopentenyl pyrophosphate (3-IPP), and they are synthesized through the mevalonate pathway in the cytoplasm or methylerythritol phosphate (MEP) pathway in the chloroplasts [6]. Most of the monoterpenes occurring in honey are derived from geranyl pyrophosphate (GPP) [1]. While monoterpenes are mostly generated in plants via the cytosolic route, norisoprenoids arise from 2-IPP and 3-IPP, derived from both MEP and mevalonate pathways [7]. Norisoprenoids may also be formed as carotenoid degradation products [8].

The most abundant monoterpenes were 4-isopropenylcyclohexa-1,3-diene-1-carboxylic acid and 4-(2-hydroxy-2-propanyl)cyclohexa-1,3-diene-1-carboxylic acid found in a particularly high percentage in linden honey (15.6% and 29.5%, respectively). The most abundant norisoprenoid was dehydrovomifoliol, found in several samples (3.6%–45.3%), and its highest percentage was found in heather honey (45.3%). Structurally related vomifoliol was abundant in eucalyptus honey (32.2%). Other relevant compounds were 3-oxo-α-ionone, most abundant in eucalyptus honey (14.2%), (*E*)-3-oxo-*retro*-α-ionol and (*Z*)-3-oxo-*retro*-α-ionol, most abundant in cornflower (7.6% and 9.4%, respectively) honey.

The identified aliphatic compounds (Table 1) included 2 aldehydes, 4 alcohols, 12 acids, and 12 hydrocarbons. The most commonly occurring were tricosane (0.6%–20.8%), (*Z*)-tricos-9-ene (0.1%–5.7%), heneicosane (0.2%–13.6%), octadecan-1-ol (0.2%–20.0%), (*Z*)-octadec-9-enoic acid (0.8%–20.8%), and octadecanoic acid (0.1%–14.7%). These compounds may derive from combs and cuticular waxes [9].

Nitrogen compounds were relatively rare within the samples and were mostly represented by various indole compounds, which biosynthetically derive from tryptophan. [10]. The mandarin honey contained 1,3-dihydro-2H-indol-2-one (4.8%), 1H-indole-2,3-dione (1.1%), and methyl indole-3-acetate (5.2%). Willow, dandelion, and red clover honey contained 2.7%, 0.9%, and 1.1% of 5-aminoindanone, respectively. The first honey also contained 1,4-dimethylindan-2-yl acetate (1.0%). The honey from *Coffea* spp. and mandarin flowers also contained methylxanthines that are synthetized from purine nucleotides [11]. Both varieties contained caffeine (56.4% and 12.5%, respectively), and the first one also contained closely related theobromine (26.5%).

Furan (12 compounds) and pyran (6 compounds) derivatives, among others, included 2,4-dihydroxy-2,5-dimethyl-3(2H)-furan-3-one (0.1%–3.0%), furan-2,5-dicarboxaldehyde (0.2%–15.4%), furyl hydroxymethyl ketone (1.0%–11.2%), 5-hydroxymethylfurfural (0.0%–1.9%), and 2,3-dihydro-3,5-dihydroxy-6-methyl-4H-pyran-4-one (0.4%–5.1%). These compounds derive from carbohydrates and can naturally form in honey as Maillard reaction products [12].

**Figure 1:**
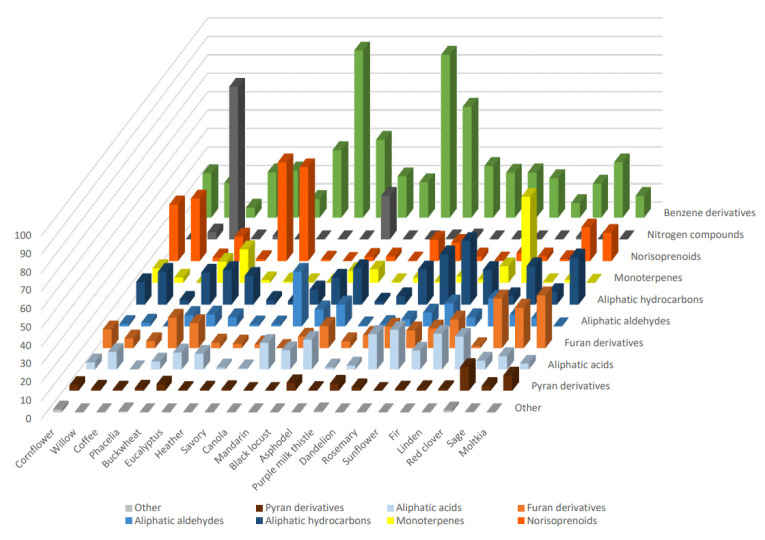
The overall percentages of the different volatile organic compounds (VOCs) structural groups in the analyzed honey types.

In summary, VOCs profiles of the investigated honey varieties are presented in Table 1 and compared in Figure 1. They were characterized by different dominant groups of compounds in terms of the percentage of the whole VOCs profile. As a high diversity can be observed among the varieties, the VOCs profile determined by DHLLE may be a useful parameter for the honey classification. Benzene derivatives dominated in savory and asphodel honeys (nearly 90%) and purple milk thistle honey (60%). Particularly rich in monoterpenes was linden honey (47%) and norisoprenoids dominated in eucalyptus, heather, willow, and cornflower honeys (54%, 51%, 34%, and 31%, respectively). Nitrogen compounds were most abundant in coffee and mandarin honeys (83% and 24%, respectively). Aliphatic aldehydes were most abundant in canola honey (30%) and, contrary to all other samples (except sunflower), they were markedly more abundant than aliphatic acids. On the other hand, aliphatic hydrocarbons constituted, respectively, 26%, 27%, and 35% of moltkia, rosemary, and sunflower honey volatile profiles. Red clover, moltkia, and sage honeys were characterized by the particular abundance of furan derivatives (22%, 27%, and 29%, respectively), and the first also contained the highest percentage of pyran derivatives (13%).

### 2.2. Comparison of the Results with Literature Data Obtained Using Ultrasonic Solvent Extraction and Solid-Phase Extraction Methods

From the practical point of view, the compounds that can be useful as markers of botanical origin of honey mostly belong to terpenes, norisoprenoids, benzene derivatives [1,13], and nitrogen-containing compounds [14]. The potential marker compounds selected for honey quality control purposes must usually be abundant enough and it must be possible to extract using the appropriate sample preparation method. As described in the previous paragraph, numerous compounds from all of these groups were extracted from different honey samples by DHLLE.

C_13_-Norisoprenoids were reported as typical compounds in a number of honey varieties, found as relevant compounds in their extracts, and they were also extracted by applying the DHLLE method. For example, dehydrovomifoliol, characteristic for eucalyptus, heather, dandelion (19.3%), and sage (up to 3.2%) honeys, as determined in USE extracts [1,15,16,17,18], in the present study represented 33.2%, 45.3%, 9.4%, and 18.2% of the volatile fraction in DHLLE extracts, respectively. Similarly, vomifoliol found in the USE extracts of willow honey (av. 24.9%) [19] was also detected in DHLLE extracts at 17.1%, and (*E*)- and (*Z*)-3-oxo-*retro*-α-ionol found in the USE extracts of cornflower (av. 9.1% and 14.4%) and phacelia (av. 5.4% and 9.4%) honey [20,21] were also identified in those obtained by DHLLE (7.6%, 9.4% and 1.8%, 2.4%, respectively). 3-Oxo-α-ionol and 3-oxo-α-ionone, typical for cornflower (3.0% and 14.1%, respectively) and eucalyptus honey [20,22] determined in extracts obtained by USE, were also found in DHLLE extracts (4.5%, 1.9% and 2.5%, 14.2%, respectively). Interestingly, the DHLLE method also enabled the detection of 3,4-dihydro-3-oxoedulan (1.4%), which is proposed as a specific marker of cornflower honey and was previously detected as a dominant compound of its headspace by headspace solid-phase microextraction (HS-SPME, up to 45.0%) [23].

Monoterpenes mostly represented a lower percentage of the honey solvent extracts. Nevertheless, exceptional amounts of linalool derivatives were found in DHLLE extracts of phacelia honey, including, among others, *trans-* and *cis-*linalool oxides (2.4% and 2.0%) and hydroxylinalool (2.6%). Previously, the average amounts of these compounds found in USE (with dichloromethane) extracts of this variety were 2.3%, 0.7%, and 2.0%, respectively [21]. A particularly high percentage of less volatile terpenic acids: 4-isopropenylcyclohexa-1,3-diene-1-carboxylic acid and 4-(2-hydroxy-2-propanyl)cyclohexa-1,3-diene-1-carboxylic acid, which are reported as markers of linden honey extracted by SPE [24], were found in the DHLLE extract of linden honey (15.6% and 29.5%) but also in smaller amounts in a few other nectar honey samples and fir honeydew honey (1.6% and 6.6%). This is in accordance with previous findings reporting slight amounts of glycosidic precursors of these compounds also in fir honeydew [25].

Several of the benzene derivatives were extracted particularly well from honeys by different methods, e.g., HS-SPME, USE, dynamic headspace extraction (DHSE), or Likens–Nickerson steam distillation/solvent extraction (SDE). Methyl syringate, a lignin derivative present in numerous honey varieties [13], may be useful as a nonspecific marker for several honey types such as savory, canola, or asphodel where it dominates. In currently analyzed samples obtained by DHLLE, the percentage of this compound reached 72.6%, 25.9%, and 85.8%, respectively. This is similar to the results obtained by USE with different solvents, when its abundance reached up to 60.1% for savory [26] and up to 87.0% for asphodel honey [27]. In addition, β-phenyllactic acid, which is considered a marker of cornflower, purple milk thistle and heather honeys, determined by GC-MS in extracts obtained by SDE, HPLC with a diode-array detector (DAD) in honey solution or by UPLC-DAD-MS/MS in SPE extracts [16,28,29], respectively, was extracted well by DHLLE (9.9%, 26.5%, and 25.6%, respectively). Similarly, some other minor compounds that are potentially useful as marker compounds were found by DHLLE: e.g., coniferyl alcohol (0.2%) previously proposed as the marker of fir honeydew honey. However, the percentage was lower than the average reported for USE with dichloromethane (3.8%) [30].

The extracts obtained by the DHLLE also enabled the detection of aliphatic acids previously found in buckwheat honey and dandelion honeys. 3-Methylbutanoic acid (3.5%), 2-methylbutanoic acid (1.5%), and pentanoic acid (0.4%) were previously reported as volatile markers in the headspace of buckwheat honey [31] detected by HS-SPME. 3-Methylbutanoic acid (2.9%), 2-methylbutanoic acid (2.8%), and 3-methylpentanoic acid (6.2%) were also found in dandelion honey by DHLLE. The literature data on the latter, depending on the used method (USE, SPE, and HS-SPME), report similar levels: 1.3–3.4%, 0.8–3.6%, and 1.7–10.6%, respectively. However, 3-methylpentanenitrile, also previously reported in this honey type by the same methods [15], was currently not found.

Furan derivatives are ubiquitous in different honey types and form *i.a.* during ripening and aging [32]. Higher levels of, e.g., furfural, dihydro-3-methyl-2(3H)-furanone, and dihydro-5-methyl-2(3H)-furanone, are, however, typical for buckwheat honey and are proposed as its markers, determined by HS-SPME [31]. These compounds, in current research, were detected only in DHLLE extracts of buckwheat honey: furfural (2.3%), dihydro-3-methyl-2(3H)-furanone (0.5%), and dihydro-5-methyl-2(3H)-furanone (4.2%). Among similar pyran compounds, maltol was found quite abundant in the extract obtained by DHLLE from red clover honey (9.3%), which is comparable to the literature data ranging from 2.6% to 20.1%, as determined in USE extracts [33].

Volatile and semi-volatile nitrogen compounds are relatively rare components of honey. Purine alkaloids were found in *Coffea* and different *Citrus* honeys. *Coffea* honey contained up to 90.5% of caffeine and up to 2.9% of theobromine [34], as found in USE extracts, and mandarin (*Citrus unshiu* Marc.) honey contained caffeine up to 7.1%, as found by USE (the highest percentage was found in dichloromethane extract) [35]. The extracts obtained by DHLLE provide similar results for caffeine (56.4% and 12.5% in coffee and mandarin honeys, respectively), but a higher level of theobromine (26.5%) was found in coffee honey using DHLLE. Considering a much different ratio of these two compounds, it may be related to its higher polarity and better extraction by more polar solvents. Other identified nitrogen compounds belonged mainly to a group of indole derivatives. Among them, 1,3-dihydro-2H-indol-2-one (4.8%), 1H-indole-2,3-dione (1.1%), and methyl indole-3-acetate (5.2%) were previously found in mandarin honey by USE/GC-MS, as well as 5-aminoindanone (2.7%) that was found in willow honey by USE/GC-MS. All these compounds were found more abundant in currently investigated DHLLE extracts than previously reported for USE extracts (1.7%, 0.9%, 5.6%, and 2.3%, respectively) [19,35].

In general, the DHLLE method enabled results comparable to those obtained with other frequently used sample preparation methods to be obtained, while reducing the sample, solvent amount, or consumption of expensive consumables. This allows more cost-efficient and environmentally friendly screenings focused on relevant marker compounds, which are valid for a wide range of honey varieties. As an example, DHLLE allowed the extraction and detection of (*E*)-3-oxo-*retro*-α-ionol (7.6%) and (*Z*)-3-oxo-*retro*-α-ionol (9.4%), 3,4-dihydro-3-oxoedulan (1.4%), 3-hydroxy-4-phenylbutan-2-one (2.1%), as well as phenyllactic acid (9.4%), from cornflower honey—all compounds have previously been reported as characteristic, and they are typical compounds for this honey type [20,23,28]. Compared to other available methods, this was achieved without the use of expensive consumables such as SPE cartridges or SPME fibers, as well as with up to a 60-fold reduction in the use of dichloromethane. On the other hand, this method may be less suitable for quantitative purposes or the investigation of the minor compounds when, e.g., SPE provides superior recoveries [22]. Nevertheless, the levels of recovery are satisfactory for fingerprinting and qualitative screening [5].

## 3. Materials and Methods

### 3.1. Materials and Samples

Analytical-grade isopropanol, dichloromethane, anhydrous MgSO_4_, and Na_2_SO_4_ were obtained from Chempur (Piekary Śląskie, Poland). The standard compounds from Table 1 were purchased from Merck (Darmstadt, Germany), Sigma Aldrich (Steinheim, Germany), or Ambinter (Orleans, France). Twenty-one selected representative samples of different unifloral honeys were used: heather (*Calluna vulgaris* (L.) Hull)*,* buckwheat (*Fagopyrum esculentum* Moench)*,* black locust (acacia) (*Robinia pseudoacacia* L.)*,* goldenrod (*Solidago* spp.)*,* canola (rapeseed) (*Brassica napus* L.)*,* fir honeydew (*Abies alba* Mill.)*,* linden (lime-tree) (*Tilia* spp.), cornflower (*Centaurea cyanus* L.), willow (*Salix* spp.)*,* coffee (*Coffea* spp.)*,* phacelia (*Phacelia tanacetifolia* Benth.), eucalyptus (*Eucalyptus* spp.), savory (*Satureja subspicata* Bartl. ex Vis.), mandarin (*Citrus unshiu* (Yu.Tanaka ex Swingle) Marcow.), asphodel (*Asphodelus microcarpus* Salzm. and Viv.), purple milk thistle (*Silybum marianum* (L.) Gaertn.), dandelion (*Taraxacum officinale* (L.) Weber ex F.H. Wigg.), rosemary (*Rosmarinus officinalis* L.), sunflower (*Helianthus annuus* L.), red clover (*Trifolium pratense* L.), sage (*Salvia officinalis* L.), and moltkia (*Moltkia petraea* (Tratt.) Griseb.). The honey samples were obtained from professional beekeepers in different parts of Croatia and Poland. The honey samples were stored at 4 °C in glass jars, in the dark. Melissopalynological analyses were performed according to the International Commission for Bee Botany [36] and confirmed the unifloral honey origin.

### 3.2. Dehydration Homogenous Liquid–Liquid Extraction Method

The sample preparation was performed as reported previously [5]. In short, an aliquot of 5 g of the honey was weighed in a 15 mL centrifuge tube, dissolved in 6 mL of ultrapure water, and 2 mL of isopropanol was added subsequently. Afterward, 6 g of MgSO_4_ was gradually added and mixed to dehydrate the sample cooled in a cold water bath. The tube was centrifuged (5 min, 3000 rpm (1107 relative centrifugal force)), which provided the separation of two phases. The upper layer containing isopropanol extract was transferred to another tube, diluted with 1 mL of dichloromethane, and washed two times with 1 mL of ultrapure water. The remaining extract was dried using anhydrous Na_2_SO_4_ and carefully concentrated under a Vigreaux column. 2 µL of the extract was used for GC-FID/MS analyses.

### 3.3. Chromatographic Conditions

The GC-FID analyses were performed using a gas chromatograph model 7890A equipped with an FID detector (Agilent Technologies, Palo Alto, CA, USA) and an HP-5MS capillary column (5% phenyl-methylpolysiloxane, 30 m, 0.25 mm i.d., coating 0.25 μm, Agilent). The GC conditions were set as previously [5]. The oven temperature was isothermal at 70 °C for 2 min, increasing from 70 to 200 °C by 3 °C·min^−1^, and held isothermally at 200 °C for another 15 min. The carrier gas was He (1.0 mL·min^−1^). The injector temperature was set to 250 °C and the FID detector temperature was 300 °C. The GC-MS analyses were performed using a similar gas chromatograph equipped with mass selective detector (MSD) model 5977E (Agilent Technologies, Palo Alto, CA, USA) and the same chromatographic conditions as for the GC-FID analysis. The MSD (electron ionization [EI] mode) was operated at 70 eV, the ion source temperature was set to 230 °C, and the mass range was 30–300 amu. The identification of the VOCs was based on the comparison of their retention indices (RI), determined relative to *n*-alkanes (C_9_–C_25_), and retention times with those reported in the literature [37], and their mass spectra with the available authentic compounds or those listed in the Wiley 9 (Wiley, New York, NY, USA) and NIST 14 (Gaithersburg, MD, USA) mass spectral libraries. The percentage composition of VOCs was calculated from the GC peak areas as the mean of the GC-FID and GC-MS analyses using the normalization method (without correction factors).

## 4. Conclusions

The tested method allowed the extraction and identification of a wide range of previously reported specific and nonspecific honey marker compounds belonging to different chemical groups (including terpenes, benzene derivatives, or nitrogen compounds). For example, the DHLLE method allowed the extraction of 3-oxo-*retro*-α-ionols and 3,4-dihydro-3-oxo-oedulan, phenyllactic acid in cornflower honey, theobromine and caffeine in coffee honey, 4-isopropenylcyclohexa-1,3-diene-1-carboxylic acid and 4-(2-hydroxy-2-propanyl)cyclohexa-1,3-diene-1-carboxylic acid in linden honey, as well as furan derivatives in buckwheat honey. The obtained results were comparable with previously reported methods, which was confirmed for a wide range of honey varieties. Considering the application of much lower volumes of very common reagents, the DHLLE method may provide economical and ecological advantages as an alternative sample preparation method. Therefore, such a methodology may be useful for the sample preparation for routine screening analyses of honey. Moreover, the comparison of chemical profiles of 21 different varietal honeys, which were obtained in the same conditions, may be useful in terms of the authenticity and traceability of varietal honeys. In further research, it would be interesting to apply the DHLLE method to other available varietal honey samples.

## Figures and Tables

**Table 1:** The volatile profiles of the dehydration homogeneous liquid–liquid extraction (DHLLE) extracts obtained from the investigated honey varieties.

	Compound	RI	Cornflower	Willow	Coffee	Phacelia	Buckwheat	Eucalyptus	Heather	Savory	Canola	Mandarin	Black locust	Asphodel	Purple milk thistle	Dandelion	Rosemary	Sunflower	Fir	Linden	Red clover	Sage	Moltkia
			Area [%]																				
	*Benzene derivatives*																						
1	1,3-Dimethylbenzene **	<900	-	-	-	-	-	-	-	1.0	-	-	-	-	-	-	-	-	-	-	-	-	-
2	1,2-Dimethylbenzene **	902	-	-	-	-	-	-	-	0.8	-	-	-	-	-	-	-	-	-	-	-	-	-
3	Benzaldehyde ^S^	966	-	-	-	0.4	-	0.4	0.1	-	-	0.3	0.7	-	-	1.1	0.3	0.8	0.4	-	-	1.1	-
4	Benzyl alcohol ^S^	1037	0.7	0.9	-	2.1	1.9	1.2	0.3	0.4	1.3	2.6	3.5	0.2	0.5	1.1	2.3	2.2	-	-	-	0.8	-
5	Phenylacetaldehyde ^S^	1048	0.4	-	0.3	0.4	0.3	0.4	0.1	0.3	-	0.6	0.9	0.9	21.0	0.2	2.9	-	-	-	2.1	14.3	0.7
6	1-Phenylethanone ^S^	1072	-	-	-	-	-	-	-	-	-	-	-	-	-	-	-	1.2	-	-	-	-	-
7	2-Phenylethanol ^S^	1116	0.6	0.9	0.1	0.5	1.0	0.3	0.1	-	-	1.0	3.9	0.1	1.0	0.2	-	-	-	-	-	-	-
8	Phenylacetonitrile ^S^	1142	-	0.1	-	-	-	-	-	-	-	-	-	-	-	-	-	-	-	-	-	-	-
9	Benzoic acid ^S^	1162	1.4	2.1	-	2.5	4.5	0.4	1.8	6.7	7.6	1.2	-	-	0.5	4.3	-	1.8	1.7	0.3	-	3.2	-
10	2,3-Dihydrobenzofuran (coumaran) ^S^	1249	1.0	0.9	0.1	3.9	4.0	-	0.3	-	3.5	4.0	3.3	-	-	2.0	-	3.8	1.2	0.7	2.4	0.5	-
11	Phenylacetic acid ^S^	1269	2.0	3.9	1.4	0.2	3.5	-	5.7	-	-	1.3	-	-	4.5	15.6	2.0	-	10.3	1.6	-	2.0	-
12	2-Methyl-5-(1-methylethyl)phenol ^S^	1309	-	-	-	-	-	-	-	-	-	-	-	-	-	-	-	-	-	-	-	0.5	-
13	4-Vinyl-2-methoxyphenol ^S^	1314	1.1	0.2	-	2.1	2.1	-	0.3	-	-	5.5	-	-	-	1.2	-	2.4	0.8	0.4	1.5	0.5	-
14	2,4,5-Trimethylphenol *	1317	0.7	-	-	-	-	0.7	1.4	-	-	-	-	-	-	0.2	-	-	-	-	-	-	-
15	Hydroquinone ^S^	1328	-	-	1.4	-	1.2	-	-	-	-	-	-	-	-	-	-	-	-	-	-	-	-
16	3-Hydroxy-4-phenylbutan-2-one ^S^	1354	2.1	1.3	1.0	1.6	-	0.4	-	-	-	-	-	-	-	-	2.4	-	-	-	-	-	-
17	Eugenol ^S^	1361	-	-	-	-	-	-	-	-	-	-	-	-	0.9	-	-	-	0.2	-	-	0.3	-
18	4-(1,1-Dimethylpropyl)phenol ^S^	1402	0.8	1.7	0.9	2.9	1.6	-	0.3	-	-	3.0	5.4	0.3	0.9	1.2	4.2	2.6	1.2	-	3.2	1.5	3.3
19	α-(Phenylmethyl)benzeneethanol ^S^	1418	3.4	-	-	-	-	-	-	-	-	-	-	-	-	-	-	-	-	-	-	-	-
20	4-(1-Methylethyl)benzoic acid (cumic acid) ^S^	1437	0.4	-	-	-	0.3	-	-	-	-	-	-	-	-	-	-	-	-	1.2	-	-	-
21	3-Phenylprop-2-enoic acid (cinnamic acid) ^S^	1445	-	-	-	-	-	-	0.5	-	-	-	-	-	-	-	-	-	-	-	-	-	-
22	4-Hydroxyphenethyl alcohol (2-(4-hydroxyphenyl)ethanol) ^S^	1445	-	1.3	-	0.5	0.9	-	-	0.4	3.3	0.7	-	-	0.2	0.5	-	0.5	1.6	1.5	-	-	-
23	Dimethyl 1,2-benzenedicarboxylate ^S^	1468	-	-	-	-	-	-	-	-	-	-	-	0.5	-	-	-	-	-	-	-	-	-
24	2,6-Bis(1,1-dimethylethyl)-4-methylphenol ^S^	1514	-	-	-	0.2	-	-	-	-	-	-	-	-	-	-	-	-	-	-	-	-	-
25	2,4-Bis(1,1-dimethylethyl)phenol ^S^	1517	-	-	-	-	0.5	0.2	0.1	0.5	0.5	0.4	-	-	-	0.3	0.6	0.3	0.2	0.5	0.8	-	-
26	Methyl-4-hydroxy-3-methoxybenzoate ^S^	1521	-	-	-	-	-	-	-	-	-	-	-	0.4	-	-	-	-	-	-	-	-	-
27	β-Phenyllactic acid ^S^	1543	9.4	-	-	-	-	-	25.6	-	-	-	-	-	26.5	-	-	-	-	-	-	-	-
28	*p-*Hydroxybenzoic acid ^S^	1575	-	1.6	-	-	3.1	-	-	-	-	-	-	-	-	-	-	-	0.5	-	-	-	7.6
29	Methyl 3,5-dimethoxybenzoate ^S^	1579	-	-	-	-	-	-	-	-	-	-	-	0.1	-	-	-	-	-	-	-	-	-
30	3,4,5-Trimethoxybenzaldehyde ^S^	1608	-	-	-	-	-	0.3	-	0.4	-	-	-	-	-	-	0.4	-	-	-	-	-	-
31	4-(4-Hydroxy-3-methoxyphenyl)butan-2-one (zingerone) ^S^	1649	-	-	-	-	-	-	-	-	-	-	-	-	-	-	-	1.7	-	-	-	-	-
32	Syringaldehyde ^S^	1665	-	-	-	1.6	-	-	-	5.9	-	-	-	-	-	-	1.3	1.0	0.4	-	2.2	-	-
33	3,4,5-Trimethoxybenzenemethanol ^S^	1667	-	-	-	-	-	-	-	1.7	-	-	-	-	-	-	-	0.8	-	-	-	-	-
34	4-Hydroxy-2-methoxycinnamaldehyde ^S^	1738	-	-	-	-	-	-	-	-	-	0.5	-	-	-	-	-	-	-	-	-	-	-
35	4-(3-Hydroxyprop-1-en-1-yl)-2-methoxyphenol ** (coniferyl alcohol)	1744	-	-	-	-	-	-	-	-	-	-	-	-	-	-	-	-	0.2	-	-	-	-
36	Methyl syringate (methyl 4-hydroxy-3,5-dimethoxybenzoate) ^S^	1774	-	3.7	-	4.7	-	5.7	-	72.6	25.9	0.7	-	85.8	4.1	-	6.2	4.7	2.6	1.6	6.1	5.2	-
37	2-Phenoxyethyl phenyl ether (1,2-diphenoxyethane) ^S^	1802	-	-	-	0.9	0.6	-	-	-	-	0.5	1.3	-	-	-	1.7	0.6	-	-	-	-	-
	*Monoterpenes*																						
38	α-Pinene ^S^	942	-	-	-	-	-	-	-	0.2	-	-	-	-	-	-	-	-	-	-	-	-	-
39	*trans*-Linalool oxide ^S^	1076	0.1	-	-	2.4	-	-	-	-	-	0.2	1.4	0.1	-	-	-	-	0.4	-	0.3	-	-
40	*cis*-Linalool oxide ^S^	1095	-	-	-	2.0	-	-	-	-	-	-	-	-	-	-	-	-	-	-	-	-	-
41	Linalool ^S^	1101	-	0.2	-	-	0.4	0.4	-	-	-	-	-	-	-	-	-	-	-	-	-	-	-
42	Hotrienol ^S^	1113	-	-	-	0.2	-	-	-	-	-	-	0.9	-	0.3	-	2.9	-	-	0.2	-	1.5	-
43	Lilac aldehyde D	1168	-	-	-	-	-	-	-	-	-	0.2	-	-	-	-	-	-	-	-	-	-	-
44	*trans*-Epoxylinalool	1178	-	-	-	0.7	-	-	-	-	-	-	-	-	-	-	-	-	-	-	-	-	-
45	*cis*-Epoxylinalool	1183	-	-	-	0.8	-	-	-	-	-	-	-	-	-	-	-	-	-	-	-	-	-
46	*p*-Cymen-8-ol ^S^	1188	0.9	-	-	-	-	-	-	-	-	-	-	-	-	-	-	-	-	-	-	-	-
47	3,7-Dimethylocta-1,5-diene-3,7-diol (terpendiol I) ^S^	1191	-	0.7	-	2.0	1.5	0.5	-	-	-	-	2.4	-	0.6	-	-	1.0	-	0.3	-	-	-
48	1,3,3-Trimethyl-2-oxabicyclo[2.2.2]octan-6-ol (2-hydroxy-1,8-cineole)	1226	-	-	-	-	-	0.2	-	-	-	-	-	-	-	-	-	-	-	-	-	-	-
49	Thymol ^S^	1298	-	-	-	0.7	0.4	0.2	0.1	-	-	0.4	0.6	0.1	0.7	0.3	0.4	0.3	0.3	-	-	-	-
50	Limonene-1,2-diol ^S^	1345	-	-	-	-	0.6	-	-	-	-	-	-	-	-	-	-	0.5	-	1.0	-	-	-
51	8-Acetoxylinalool	1349	-	-	-	-	1.1	-	-	-	-	-	-	-	-	-	-	-	-	-	-	-	-
52	Hydroxylinalool **	1365	-	0.7	-	2.6	-	-	-	-	-	5.5	1.9	-	1.0	0.4	-	-	-	-	-	-	-
53	*p*-Menth-1-ene-7,8-diol ^S^	1463	-	1.2	-	-	-	-	-	-	-	-	-	-	-	-	-	-	-	-	-	-	-
54	4-Isopropenylcyclohexa-1,3-diene-1-carboxylic acid	1531	5.3	-	-	-	5.0	-	0.5	-	-	-	-	-	-	-	-	-	1.6	15.6	-	-	-
55	4-(2-Hydroxy-2-propanyl)cyclohexa-1,3-diene-1-carboxylic acid	1611	1.5	-	-	-	9.1	-	-	-	-	-	-	-	-	-	-	-	6.6	29.5	-	-	-
	*Norisoprenoids*																						
56	4-Ketoisophorone (3,5,5-trimethyl-cyclohex-2-ene-1,4-dione) ^S^	1147	0.2	0.2	-	-	-	0.5	-	-	-	-	-	-	-	-	-	-	-	-	-	-	-
57	2,2,6-Trimethylcyclohexane-1,4-dione ^S^	1171	0.2	-	-	-	-	0.1	0.1	-	-	-	-	-	-	-	-	-	-	-	-	-	-
58	4-Hydroxy-3,5,5-trimethylcyclohex-2-en-1-one ^S^	1312	-	-	-	-	-	1.4	-	-	-	-	-	-	-	-	-	-	-	-	-	-	-
59	β-Damascenone ^S^	1385	-	0.3	-	-	-	-	0.1	-	-	-	-	-	-	-	-	-	-	-	-	-	-
60	3,4-Dihydro-3-oxoedulan	1488	1.4	-	-	-	-	-	-	-	-	-	-	-	-	-	-	-	-	-	-	-	-
61	3-Hydroxy-β-damascone	1617	-	2.8	-	-	0.1	-	-	-	-	-	-	-	-	0.5	-	-	-	-	-	-	-
62	3-Oxo-α-damascone	1642	-	2.0	-	-	-	-	2.1	-	-	-	-	-	-	-	-	-	-	-	-	-	-
63	3-Hydroxy-7,8-dihydro-β-ionol	1650	-	1.0	-	-	-	-	-	-	-	-	-	-	-	-	-	-	-	-	-	-	-
64	3-Oxo-α-ionol (4-(3-hydroxy-but-1-enyl)-3,5,5-trimethyl-2-cyclohexen-1-one) ^S^	1660	4.5	-	-	1.0	-	2.5	1.3	-	-	-	-	0.1	1.9	-	-	-	0.5	-	-	-	-
65	3-Oxo-α-ionone (3,5,5-trimethyl-4-(3-oxo-but-1-enyl)-2-cyclohexen-1-one) ^S^	1665	1.9	-	0.5	1.5	-	14.2	1.0	-	-	-	-	-	4.5	-	-	-	-	-	-	-	-
66	3-Hydroxy-5,6-epoxy-β-ionone	1689	-	-	-	-	-	-	-	-	-	-	-	-	-	-	-	-	-	-	0.9	-	-
67	3-Oxo-7,8-dihydro-α-ionone	1720	-	-	-	1.3	-	0.7	-	-	-	-	-	-	-	-	-	-	-	-	-	-	-
68	(*E*)-3-Oxo-*retro*-α-ionol (6,7-dehydro-7,8-dihydro-3-oxo-alpha-ionol)	1734	7.6	1.0	-	1.8	-	0.8	1.0	-	-	-	-	-	-	-	-	-	-	-	-	-	-
69	(*Z*)-3-Oxo-*retro*-α-ionol (9-(hydroxymegastigma-4,6-dien-3-one)	1787	9.4	7.6	-	2.4	-	0.1	-	-	-	-	-	-	-	-	-	-	-	-	-	-	-
70	4-hydroxy-3,5,6-trimethyl-4-(3-oxo-1-butenyl)cyclohex-2-en-1-one	1790	-	1.9	1.3	-	-	-	-	-	-	-	-	-	-	-	-	-	-	-	-	-	-
71	Dehydrovomifoliol ^S^	1796	-	-	-	-	-	-	45.3	-	-	-	-	-	-	9.4	-	-	3.6	-	-	18.2	-
72	Vomifoliol ^S^	1802	5.4	17.1	-	4.5	0.9	33.2	-	0.8	-	2.2	2.4	-	5.0	-	2.3	0.2	-	2.4	-	-	15.2
73	(*E*)-4-r-1′,t-2′,c-4′-Trihydroxy-3′,6′,6′-trimethylcyclohexyl)but-3-en-2-one	1949	-	-	-	0.7	-	-	-	-	-	-	-	-	-	-	-	-	-	-	-	-	-
	*Aliphatic aldehydes*																						
74	3-Methylpentanal ^S^	1022	-	-	-	-	1.0	-	0.1	-	-	-	-	-	-	-	-	-	-	-	-	-	-
75	Nonanal ^S^	1105	-	-	-	-	-	-	-	-	-	0.3	-	-	-	-	-	-	-	-	-	-	-
	*Aliphatic alcohols*																						
76	Dodecan-1-ol ^S^	1479	1.3	-	-	2.5	2.9	1.2	0.3	-	-	1.0	6.3	-	-	2.1	2.1	2.8	1.8	-	3.4	1.2	-
77	Hexadecan-1-ol ^S^	1882	-	-	-	0.4	0.5	0.4	-	-	1.2	0.9	0.7	-	-	-	0.8	0.6	0.8	-	0.6	-	-
78	(*Z*)-Octadec-9-en-1-ol ^S^	2060	-	-	-	2.1	1.1	2.1	-	0.6	8.4	4.5	2.8	0.2	0.7	1.1	2.8	5.5	2.3	6.7	1.2	2.4	-
79	Octadecan-1-ol ^S^	2084	-	1.9	-	1.1	0.9	1.2	0.7	0.5	20.0	2.4	2.0	0.2	0.5	0.6	1.8	3.5	-	8.3	1.2	1.0	-
	*Aliphatic acids*																						
80	3-Methylbutanoic acid (isovaleric acid) ^S^	<900	-	0.2	-	-	3.5	7.6	-	-	-	-	-	-	-	2.9	-	-	-	-	-	-	-
81	2-Methylbutanoic acid ^S^	<900	-	0.2	-	0.1	1.5	-	-	-	-	-	-	-	-	2.8	-	-	-	-	-	-	-
82	Pentanoic acid (valeric acid) ^S^	<900	-	-	-	-	0.4	-	-	-	-	-	-	-	-	-	-	-	-	-	-	-	-
83	3-Methylpentanoic acid (3-methylvaleric acid ^S^	952	-	1.0	-	-	-	-	0.0	-	-	-	-	-	-	6.2	-	-	-	-	-	-	-
84	(*E*)-2-methylpent-2-enoic acid ^S^	1006	-	-	-	-	0.5	-	-	-	-	-	-	-	-	-	-	-	-	-	-	-	-
85	2-Ethylhexanoic acid ^S^	1133	-	-	-	-	-	-	0.2	-	-	-	-	-	-	-	-	-	-	-	-	-	-
86	Nonanoic acid ^S^	1283	-	-	-	-	-	1.0	-	-	-	-	-	-	-	-	-	-	-	-	-	-	-
87	Dodecanoic acid ^S^	1573	-	-	-	-	-	-	-	-	-	-	-	-	-	0.4	-	2.1	1.0	-	-	-	-
88	Tetradecanoic acid ^S^	1769	-	-	-	-	-	-	-	-	-	-	-	-	-	-	-	0.8	-	-	-	-	-
89	Hexadecanoic acid ^S^	1963	-	1.0	-	1.2	2.4	-	-	0.5	-	-	-	-	-	-	-	2.0	2.2	1.6	0.9	-	-
90	(*Z*)-Octadec-9-enoic acid ^S^	2147	3.2	3.1	-	2.1	1.0	-	0.8	-	-	9.5	14.1	0.9	1.6	5.8	20.8	2.5	13.9	14.7	1.8	7.3	-
91	Octadecanoic acid ^S^	2181	0.7	4.1	-	0.9	-	-	-	-	14.7	1.1	2.3	0.1	0.5	1.3	1.0	3.0	2.6	1.8	2.1	-	3.2
	*Aliphatic hydrocarbons*																						
92	Dodecane ^S^	1200	0.2	0.4	-	2.0	-	0.3	-	-	-	0.4	-	0.1	0.2	-	0.8	0.5	-	-	-	-	-
93	Tetradecane ^S^	1400	0.9	1.0	0.5	1.8	1.1	0.5	0.3	-	-	1.0	3.0	0.2	0.6	0.7	1.4	1.1	0.7	-	1.7	1.0	1.7
94	Pentadecane ^S^	1500	-	-	-	-	0.5	0.3	-	-	-	-	-	0.1	-	-	-	0.5	-	-	-	-	-
95	Hexadecane ^S^	1600	-	-	0.4	1.3	0.8	1.2	-	-	-	0.5	1.1	0.1	-	0.6	1.0	0.8	0.5	-	1.3	1.2	-
96	Heptadecane ^S^	1700	-	-	-	-	0.4	-	-	-	-	-	-	-	-	-	-	0.8	0.3	-	1.0	-	-
97	Octadecane ^S^	1800	-	-	-	-	-	-	-	-	-	-	-	-	-	-	-	1.3	0.6	-	1.6	-	-
98	Nonadecane ^S^	1900	-	-	-	-	-	0.2	-	-	-	0.3	-	0.1	-	-	0.4	0.4	0.6	-	0.2	-	-
99	Eicosane ^S^	2000	-	-	-	-	0.6	0.2	-	-	-	-	-	-	-	0.5	0.9	4.5	2.3	-	-	-	-
100	Heneicosane ^S^	2100	0.7	4.1	0.5	2.5	1.0	-	0.5	-	-	2.5	4.3	0.2	0.3	9.9	13.6	2.0	2.1	2.1	3.0	0.9	4.5
101	(*Z*)-Tricos-9-ene	2265	-	1.1	-	2.5	1.1	1.4	0.2	1.7	-	4.1	5.7	0.1	0.3	0.7	2.1	1.8	2.2	0.9	2.5	0.9	2.3
102	Tricosane ^S^	2300	3.4	11.1	1.1	7.1	13.2	5.5	1.4	-	8.0	5.8	5.5	0.6	3.0	6.6	7.0	20.8	9.5	1.5	9.5	2.7	17.0
103	Tetracosane ^S^	2400	6.9	-	-	-	-	5.9	-	-	-	-	-	-	-	-	-	-	-	-	-	-	-
	*Nitrogen compounds*																						
104	1,3-Dihydro-2H-indol-2-one ^S^	1480	-	-	-	-	-	-	-	-	-	4.8	-	-	-	-	-	-	-	-	-	-	-
105	5-Aminoindanone ^S^	1594	-	2.7	-	-	-	-	-	-	-	-	-	-	-	0.9	-	-	-	-	1.1	-	-
106	1,4-Dimethylindan-2-yl acetate	1651	-	1.0	-	-	-	-	-	-	-	-	-	-	-	-	-	-	-	-	-	-	-
107	1H-Indole-2,3-dione ^S^	1724	-	-	-	-	-	-	-	-	-	1.1	-	-	-	-	-	-	-	-	-	-	-
108	1H-Indole-3-carboxaldehyde ^S^	1824	-	-	-	-	1.7	-	-	-	-	-	-	-	-	-	-	-	-	-	-	-	-
109	Caffeine ^S^	1856	-	-	56.4	-	-	-	-	-	-	12.5	-	-	0.5	-	-	-	-	-	-	-	-
110	Theobromine ^S^	1925	-	-	26.4	-	-	-	-	-	-	-	-	-	-	-	-	-	-	-	-	-	-
111	Methyl indole-3-acetate ^S^	1995	-	-	-	0.3	-	-	-	-	-	5.2	-	-	-	-	-	-	-	-	-	-	-
	*Furan derivatives*																						
112	Furfural ^S^	<900	-	-	-	-	2.3	-	-	-	-	-	-	-	-	-	-	-	-	-	-	-	-
113	Furyl alcohol ^S^	<900	-	-	-	-	1.0	-	-	-	-	-	-	-	-	-	-	-	-	-	-	-	-
114	1-(2-Furanyl)ethanone (2-acetylfuran) ^S^	908	-	-	-	-	0.2	-	-	-	-	-	-	-	-	-	-	-	-	-	-	-	-
115	Dihydro-3-methyl-2(3H)-furanone (α-methyl-γ-butyrolactone) ^S^	955	-	-	-	-	0.5	-	-	-	-	-	-	-	-	-	-	-	-	-	-	-	-
116	Dihydro-5-methyl-2(3H)-furanone (γ-valerolactone) ^S^	964	-	-	-	-	4.2	-	-	-	-	-	-	-	-	-	-	-	-	-	-	-	-
117	5-Methyl-2-furancarboxaldehyde ^S^	969	0.4	-	-	-	-	-	-	-	-	-	-	0.1	0.4	-	-	-	-	-	-	-	-
118	2,4-Dihydroxy-2,5-dimethyl-3(2H)-furan-3-one	982	1.0	-	0.4	1.0	0.9	0.7	0.2	0.4	-	1.1	1.8	0.1	0.8	0.9	0.8	0.9	0.8	1.0	-	0.5	3.0
119	3-Hydroxy-4,4-dimethyldihydro-2(3H)-furanone (pantolactone) ^S^	1043	-	-	-	-	-	-	-	-	-	-	-	-	-	-	-	-	1.3	-	-	-	-
120	Furan-2,5-dicarboxaldehyde ^S^	1084	3.6	1.9	1.6	15.4	0.2	1.1	1.0	-	-	2.0	1.7	0.9	0.5	7.9	8.7	6.4	8.1	-	15.2	10.5	13.6
121	Furyl hydroxymethyl ketone ^S^	1086	4.5	3.0	1.6	-	4.3	1.1	1.0	1.4	-	2.7	7.6	1.8	4.1	2.7	-	3.3	4.7	-	10.6	9.0	11.2
122	Dihydro-4-hydroxy-2(3H)-furanone ^S^	1193	-	-	-	-	-	-	-	-	-	-	-	0.1	-	-	-	-	-	-	-	-	-
123	5-Hydroxymethylfurfural ^S^	1230	0.7	0.4	0.2	0.1	0.0	0.0	0.2	0.1	-	0.4	0.9	0.7	1.3	0.2	0.1	0.1	0.7	0.0	1.1	1.9	0.7
	*Pyran derivatives*																						
124	Maltol (3-hydroxy-2-methyl-4H-pyran-4-one) ^S^	1117	-	-	-	-	-	-	-	-	-	-	-	-	-	-	-	-	-	-	9.3	-	3.3
125	2,3-Dihydro-3,5-dihydroxy-6-methyl-4H-Pyran-4-one ^S^	1151	2.6	1.1	0.8	1.3	2.7	0.4	0.6	0.9	-	-	4.3	0.4	3.9	1.9	-	0.8	0.9	0.9	3.8	2.6	5.1
126	3,5-Dihydroxy-2-methyl-4H-pyran-4-one ^S^	1192	0.6	-	-	-	0.8	-	-	-	-	-	-	-	-	-	-	-	-	-	-	-	-
	*Other*																						
127	2-Hydroxycyclopent-2-en-1-one ^S^	934	1.1	-	-	-	-	-	-	-	-	-	-	-	-	-	-	-	-	-	-	-	-
128	2-Hydroxy-3-methylcyclopent-2-en-1-one (corylone) ^S^	1033	0.3	-	-	-	-	-	-	-	-	-	-	0.0	0.4	-	-	-	-	-	-	0.1	-
129	4-Methyl-4-vinylbutyrolactone (lavender lactone)	1042	-	-	-	0.4	-	-	-	-	-	-	-	-	-	-	-	-	-	-	-	-	-
130	1,1,6-Trimethyl-1,2-dihydronaphthalene (dehydro-ar-ionene) ^S^	1354	-	-	-	-	-	-	-	-	-	-	-	-	-	-	-	-	-	-	1.0	-	-

*—Tentatively identified, **—Correct isomer not identified; RI—retention indices determined relative to n-alkanes (C_9_–C_25_) on the HP-5MS column; ^S^—identification confirmed with standard compound.

## Sample Availability

Samples are available from the authors for limited time.
